# Remote Sensing of Dispersed Oil Pollution in the Ocean—The Role of Chlorophyll Concentration

**DOI:** 10.3390/s21103387

**Published:** 2021-05-13

**Authors:** Kamila Haule, Włodzimierz Freda

**Affiliations:** Department of Physics, Gdynia Maritime University, ul. Morska 81-87, 81-225 Gdynia, Poland; w.freda@wm.umg.edu.pl

**Keywords:** radiative transfer, oil pollution, dispersed oil detection, remote sensing reflectance, chlorophyll-a, color index

## Abstract

In the contrary to surface oil slicks, dispersed oil pollution is not yet detected or monitored on regular basis. The possible range of changes of the local optical properties of seawater caused by the occurrence of dispersed oil, as well as the dependencies of changes on various physical and environmental factors, can be estimated using simulation techniques. Two models were combined to examine the influence of oceanic water type on the visibility of dispersed oil: the Monte Carlo radiative transfer model and the Lorenz–Mie model for spherical oil droplets suspended in seawater. Remote sensing reflectance, *R_rs_*, was compared for natural ocean water models representing oligotrophic, mesotrophic and eutrophic environments (characterized by chlorophyll-a concentrations of 0.1, 1 and 10 mg/m^3^, respectively) and polluted by three different kinds of oils: biodiesel, lubricant oil and crude oil. We found out that dispersed oil usually increases *R_rs_* values for all types of seawater, with the highest effect for the oligotrophic ocean. In the clearest studied waters, the absolute values of *R_rs_* increased 2–6 times after simulated dispersed oil pollution, while *R_rs_* band ratios routinely applied in bio-optical models decreased up to 80%. The color index, CI, was nearly double reduced by dispersed biodiesel BD and lubricant oil CL, but more than doubled by crude oil FL.

## 1. Introduction

In near-shore areas, the majority of oil products that enter the sea because of human activity come from typically small but frequent and widespread releases, such as river inflows, containing industrial and agricultural runoff, or daily shipping activities [1,2]. On the other hand, open ocean oil pollution is usually caused by oil spills (whether accidental or operational) [3]. Dispersed oil in the water column is one of the long-term results of oil spilled on the ocean surface caused both by natural dispersion induced by wave action [4,5] and chemical dispersant treatments e.g., [6,7,8]. While natural dispersion occurs within days to months after an oil spill depending on oil type, wave action and some environmental conditions [5], chemically induced oil dispersion occurs much faster, usually within hours to days after spillage [9,10]. In both cases, the process of dispersion occurs in a vertical direction and it involves oil droplets of sizes smaller than ~100 μm [2].

Dispersed oil pollution is no less a threat to the marine life than oil spilled on the sea surface. It affects filter-feeding organisms and their primary consumers e.g., [11,12,13], sea bottom fauna and their consumers [14,15] as well as plankton composition [16,17]. As a result, it reduces biodiversity and consequently accelerates the eutrophication process [18,19]. Despite the widely researched spilled oil including in situ, ship-borne, airplane and space-borne techniques [20,21], the fates of dispersed oil in seawater are much less known and are not monitored on a regular basis. Recently dispersed oil gained attention in marine research; however, most studies focus on its biological and ecological impact [22,23], chemical and microbiological consequences [24,25,26] or on oil spill modeling [27,28,29]. Few studies focus on optical properties of dispersed oil demonstrating the significance and potential utility of optical sensing of oil droplets using both active and passive remote techniques [30,31,32,33].

Remote sensing of dispersed oil is not yet practiced, although satellite and airborne remote assessment of surface oil slicks has been practiced for many years [21,34,35]. Techniques commonly applied to monitor oil slicks, such as microwave radars, laser fluorosensors or radiometers, have never demonstrated their applicability to dispersed forms of oil. One reason is the lack of sufficient technical possibilities; another is the lack of appropriate models and algorithms. The first reason is likely to be removed as the new generations of satellite and airborne sensors become more sensitive and have wider spectral ranges. Nevertheless, there is still need for methods and algorithms to provide quantitative assessments of the dispersed oil products remaining in seawater. Quantification of dispersed oil pollution can enable corresponding preventive and clean-up actions.

On the other hand, the calibration of satellite-based algorithms requires understanding of the empirical relationships between remote sensing reflectance *R_rs_* and inherent optical properties (IOPs) of seawater [36], which can be very specific in complex waters. Oil droplets participate in the process of radiative transfer in seawater as optically significant components [37,38]. They absorb and scatter light; therefore, they affect both the inherent and the apparent optical properties in the area of their occurrence [39]. The scope of their influence can be estimated using numerical modeling based on the radiative transfer equation, as long as their IOPs are known [40,41]. The IOPs of dispersed oils can be obtained by application of Lorenz–Mie theory for spherical particles suspended in a non-absorbing medium [42,43]. Numerical modeling is a convenient tool for understanding the impact of specific seawater components on the light field. It is widely used in oceanography in order to perform large-scale analyses where direct measurements cannot be performed or cannot provide satisfying information e.g., [44,45,46,47]. Successful application of radiative transfer for seawater polluted by dispersed oil droplets opened a source of valuable data for understanding the impact of various factors on the visibility (potential detectability) of dispersed oil [48]. It also became a tool for designing field experiments and creating future algorithms for remote detection of dispersed oil [49].

Optical properties of open ocean waters are determined mostly by chlorophyll-a concentration (*Chl-a*), contrary to complex coastal waters [50]. Ocean waters are frequently classified because of their trophic status in terms of *Chl-a* concentration: oligotrophic waters of *Chl-a* ≤ 0.1 mg/m^3^, mesotrophic waters of 0.1 < *Chl-a* ≤ 1.67 mg/m^3^ and eutrophic waters of *Chl-a* > 1.67 mg/m^3^ [51]. This study is focused on finding dependencies between chlorophyll concentration and the impact of three kinds of dispersed oils characterized by different optical properties: crude oil *Flotta*, cylinder lubricant *Cyliten N460* and biodiesel *BIO-100*, on the remote sensing reflectance *R_rs_* in open ocean waters. Furthermore, we analyze the influence of dispersed oils on frequently used reflectance band ratios and band differences and demonstrate their utility in future algorithms for dispersed oil detection.

## 2. Materials and Methods

### 2.1. Optical Model of Oceanic Water

**Figure 1:**
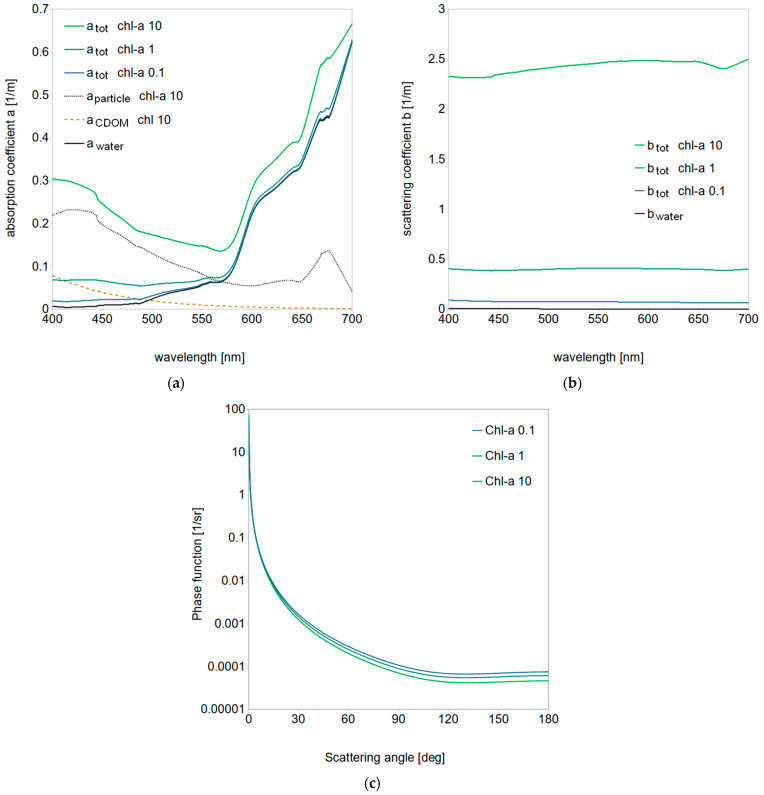
Inherent optical properties for oceanic water model characterized by chlorophyll-a concentrations of 0.1, 1 and 10 mg/m^3^: (**a**) total absorption coefficients a_tot_ for three types of oceanic waters and the absorption contribution coming from chlorophyll particles, a_particle_, color-dissolved organic matter, a_CDOM_, and pure water, a_water_, for chl-a of 10 mg/m^3^; (**b**) total scattering coefficients for three types of oceanic waters b_tot_ and for pure water b_water_; (**c**) log-linear plot of corresponding scattering phase functions at 555 nm.

Radiative transfer simulations were conducted for representative types of oligotrophic, mesotrophic and eutrophic oceanic waters characterized by corresponding chlorophyll-a concentrations of 0.1, 1 and 10 mg/m^3^. The model of natural seawater includes the IOPs of three components: pure water, chlorophyll particles and color-dissolved organic matter (CDOM), plotted in Figure 1. Pure water spectral coefficients of absorption a_water_ were adapted from Pope and Fry [52] (solid black line in Figure 1a) and spectral coefficients of scattering b_water_ from Morel [53,54] (solid black line in Figure 1b). Chlorophyll-a and CDOM absorption coefficients, a_particle_ and a_CDOM_, respectively, were calculated according to the formulas proposed by Bricaud and Mobley [54,55]. Particle scattering coefficients b_particle_ were chosen after Loisel and Morel [56] with updates of Morel [57]. Figure 1a shows the total absorption coefficients of all three types of considered ocean waters as well as their three components for *Chl-a* = 10 mg/m^3^. Figure 1b illustrates the total scattering coefficients of all ocean water types and pure water. Scattering phase functions for oceanic waters were calculated after Morel [57] as a function of chlorophyll-a concentration and wavelength and are presented in Figure 1c on a log-linear plot. In this study we simplified the model by assuming a constant vertical concentration of *Chl-a*.

### 2.2. Optical Model of Dispersed Oil

The study was conducted for three kinds of oil:

1. Biodiesel *BIO-100* (hereafter referred to as BD), commercially available at PKN Orlen. It consists of over 96% of fatty acid methyl esters, and it is the most common type of biodiesel in Europe. The fuel is made from vegetable oils and can be used in most diesel engines [58,59].
2. Cylinder lubricant oil *Cyliten N460* (CL), commercially available at LOTOS S.A., formulated upon deeply refined, dewaxed and hydrorefined mineral oils (>80%) with low susceptibility to coking, and greased with vegetable oil (<20%) to improve lubrication properties [60]. It is designed for lubrication of high-pressure compressors and other special applications as low-speed gears, e.g., in marine ship engine systems.
3. Crude oil *Flotta* (FL), extracted offshore in the North Sea in the British exclusive economic zone. It is a mixture of thousands of hydrocarbons of paraffin-naphthene base [2,61], characterized by an API gravity of 36.6, total sulfur content of 0.66% wt and total wax content of 6.75% wt.

The IOPs of dispersed oil cannot be measured directly due to absorption values below the detection limit of commercially available spectrometers; therefore, they were calculated using Lorenz–Mie theory for spherical particles suspended in a non-absorbing medium (pure water) with correction for the saline water absorption [43]. Input data for Mie modeling include a spectrum of the complex refractive index of light in pure oil and oil droplet size distribution.

**Figure 2:**
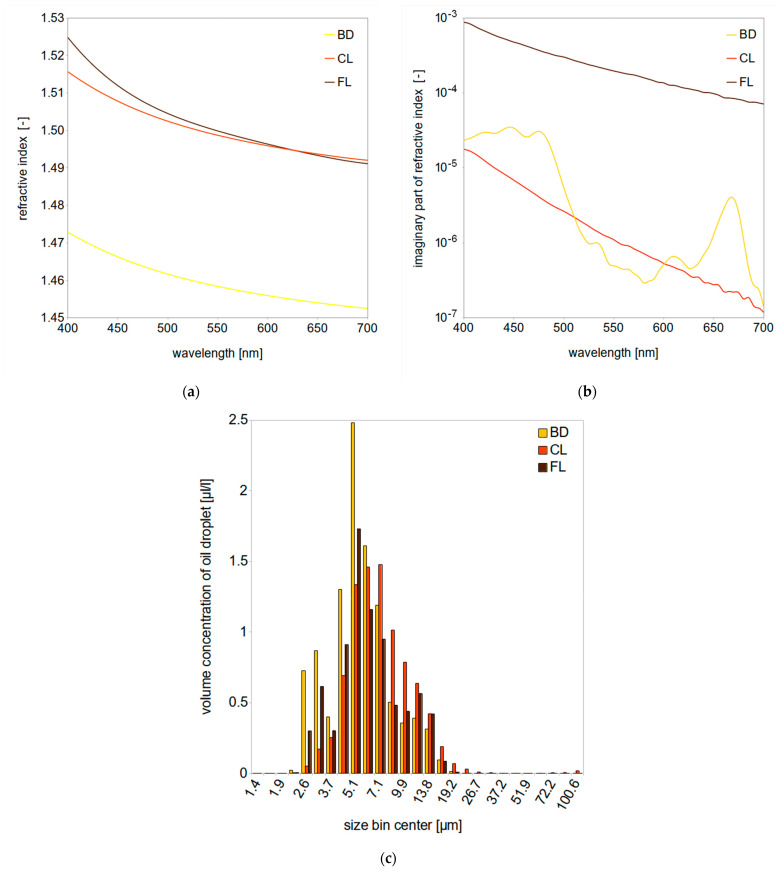
(**a**) Real part of refractive indices of oils measured using multispectral refractometer DSR-λ; (**b**) imaginary part of refractive indices of oils obtained from absorption measurements by spectrophotometer Perkin Elmer Lambda 850; (**c**) oil droplet size distributions measured using LISST-100X.

The real parts of refractive indices of original oils for this study were measured using an automatic critical-angle dispersion multi-wavelength refractometer DSR-λ (2010, Schmidt + Haensch GmbH & Co, Berlin, Germany) for nine wavelengths in the VIS range at 20 °C according to the methodology from [62]. Droplets of oil were assumed to be suspended in natural saline water (i.e., medium) characterized by refractive indices given by [63] for salinity of 35 PSU and temperature of 20 °C. Obtained multispectral data were further approximated by fourth-order polynomial functions, plotted in Figure 2a. Imaginary parts of the complex refractive index of light for pure oils are shown in Figure 2b. They were calculated from absorption measurements performed with the use of Perkin Elmer Lambda 850 dual-beam spectrophotometer (PerkinElmer Inc., Waltham, MA, USA) equipped with a 15-cm integrating sphere (Labsphere Inc., North Sutton, NH, USA) according to the methodology described in [64]. Absorption measurements were collected within the spectral region of visible light from 400 to 700 nm at 1-nm intervals. 

To further take a step forward compared to previous studies [37,38,40,48], we applied droplet size distributions derived from measurements conducted using LISST-100X in the stationary mode. Oil-in-water dispersions were prepared in the laboratory by means of mechanical mixing. First, selected oils were homogenized in demineralized water in the volume concentration of (2 ± 0.3) × 10^3^ μL/L using a laboratory homogenizer MPW-120 (run parameters: time—10 min, rotation speed—10,000 rps, giving the energy dissipate rate per volume of 2.083 × 10^5^ J/m^3^s.). In the second step, oil dispersions were diluted in demineralized water to the final volume concentration of 10 ppm. This procedure ushered the way to obtain stable oil-in-water dispersions which were stored in glass bottles. All droplet size distributions were obtained every 3 s by operating the LISST-100X in real-time operation mode. Each measurement comprised the average of min 100 scans and was repeated 3 times for each oil sample in order to minimize potential heterogeneity uncertainties. In this study, we used size distributions for dispersed oils collected on the day of preparation. The results are plotted in Figure 2c.

### 2.3. Radiative Transfer Simulation Setup

Radiative transfer simulations were conducted using the Monte Carlo code created and made available by Prof. Jacek Piskozub [65] and applied also by the authors of [66,67,68,69,70]. We adapted the model for studies of remote sensing of dispersed oil in seawater described previously in [39,40,49].

**Figure 3:**
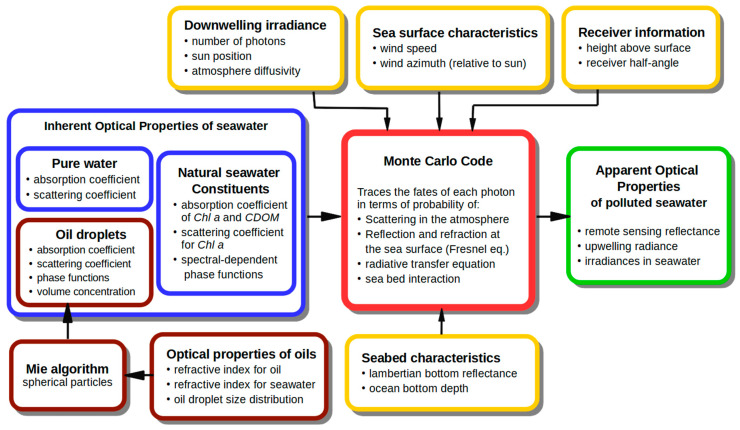
Scheme of the model of radiative transfer in seawater polluted by dispersed oil: yellow boxes mark the input boundary conditions, blue boxes illustrate the input optical properties of natural seawater; data related to dispersed oil droplets are in brown boxes, and model output data are in the green box.

Figure 3 shows the model scheme, where blue boxes indicate the inherent optical properties of oceanic water constituents (described in Section 2.1 of this paper), brown boxes display data related to dispersed oil (described in Section 2.2), yellow boxes frame the boundary weather and sea bottom conditions (described in this section below) and the green box illustrates model output data. The output remote sensing reflectance *R_rs_* is the water-leaving radiance (*L_w_*, W m^−2^ nm^−1^ sr^−1^) normalized by the downwelling irradiance (*E_d_*, W m^−2^ nm^−1^) just above the sea surface (0^+^).

**Figure 4:**
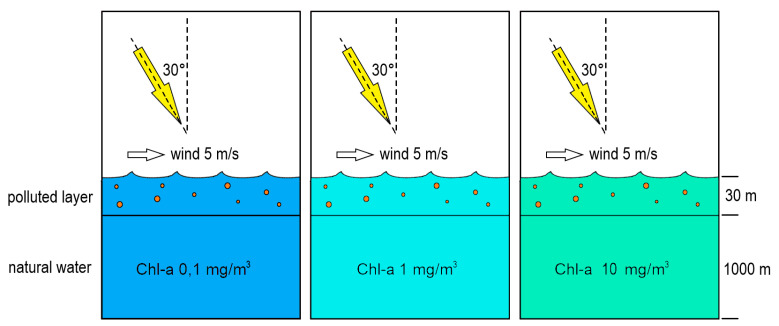
The modeling concept and boundary conditions setup in three types of oceanic waters.

The experiment design is visualized in Figure 4. All simulations were conducted for 27 wavelengths from visible light range (400–700 nm), typical for the most common remote sensors. The Monte Carlo simulation was set to trace the pathways of 2 × 10^9^ photons at each wavelength. Calculations were performed for clear sky conditions with sun height of 30° (from zenith) and the wavelength-dependent atmosphere diffusivity was averaged from the available data for low and medium atmosphere turbidity after [71]. 

Ocean depth was set at 1000 m. Ocean bottom was assumed to be of Lambertian type with 2% mirror reflection and 8% diffusive reflection; however, the depth of 1000 m ensured that the bottom parameters did not affect the *R_rs_* results. The sea surface was parameterized by the wave slope Cox–Munk distribution for a gentle wind of 5 m/s. The simulated receiver half-angle was 3.5°, which is typical for commercially available radiometers (e.g., Ramses, TriOS GmbH, Rastede, Germany). The receiver was placed just above sea surface (0^+^). Dispersed oil was assumed to be present in the surface mixed layer of 30 m in constant concentration of 1 ppm (part per million).

## 3. Results

The results of our two-step modeling series include Mie calculations of the inherent optical properties of three kinds of dispersed oils, as well as their further involvement in the radiative transfer simulation for three types of oceanic seawater characterized by *Chl-a* concentrations of 0.1, 1 and 10 mg/m^3^.

### 3.1. Dispersed Oil Optical Properties

So far, there are few complete datasets for Mie modeling of dispersed oils. Examples of such data are those collected in the 1990s [72,73], that were made using a self-adapted spectrophotometer for two kinds of crude oils characterized by extremely different optical properties: *Petrobaltic* and *Romashkino*. In this study we applied new data from direct measurements for three different types of oil: biodiesel BD, lubricant oil CL and crude oil FL. All oils have the real part of the refractive index of 1.45–1.53 (see Figure 2a), higher than seawater (~1.34).

**Figure 5:**
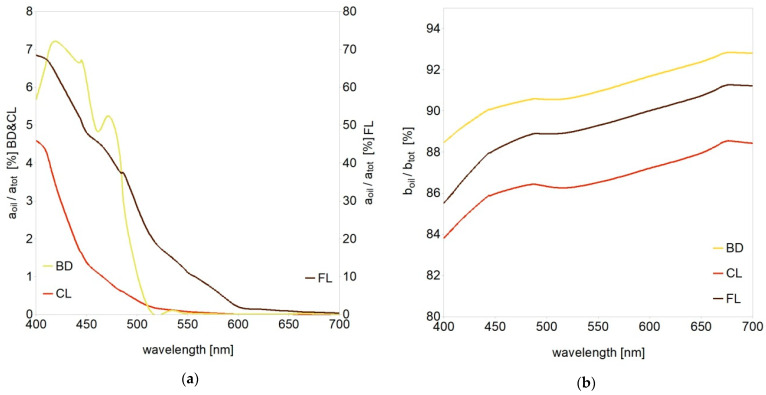
Percentage impact of (**a**) absorption and (**b**) scattering coefficients of dispersed oils to the total absorption and scattering of oligotrophic ocean water (*Chl-a* = 0.1 mg/m^3^) obtained as a result of Mie modeling.

As a result of Mie modeling, we obtained the IOPs of each kind of oil dispersions. They include absorption and scattering coefficients for 27 wavelengths from the visible spectral range (400–700 nm) and angular phase functions of the volume scattering function in the same spectral range. Figure 5a shows the impact of dispersed oil absorption to the total oligotrophic ocean water absorption (*Chl-a* = 0.1 mg/m^3^), expressed in %. The impact of dispersed crude oil FL was about 10 times higher than for dispersed BD and CL. This is because the absorption coefficient of the dispersed FL was about two orders of magnitude higher than for the other oils. Moreover, FL and CL had a similar shape of absorption spectrum, decreasing exponentially with increasing wavelengths (see Figure 2b). The absorption spectrum of BD had a different polynomial shape with three local maxima. The impact of dispersed oil to the oceanic absorption was the most significant in the blue to green spectral range. The maximal impact varied from 0.3% for CL, 0.4% for BD and 12% for FL for the eutrophic ocean, reaching up to 4.6% for CL, 7.3% for BD and 68% for FL for the oligotrophic ocean.

**Figure 6:**
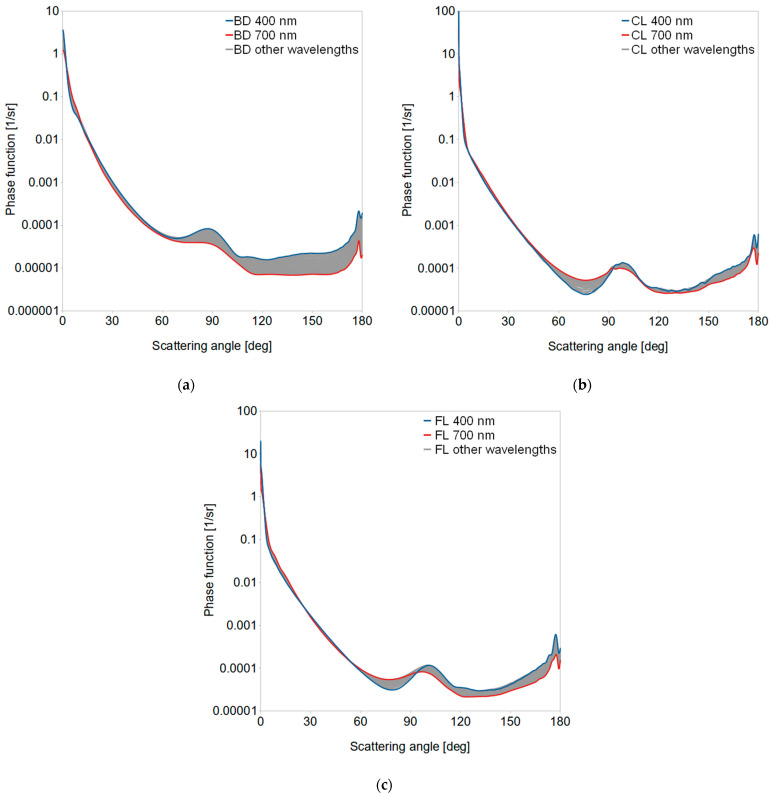
Scattering phase functions from Mie calculations for three kinds of dispersed oils: (**a**) biodiesel BD, (**b**) lubricant oil CL and (**c**) crude oil FL, within the borders of visible spectral range (400 and 700 nm).

Figure 5b similarly illustrates the impact of dispersed oils to the total scattering coefficient of oligotrophic water. It reached up to 88% for CL, 91% for FL and almost 93% for BD. However, for eutrophic ocean it was much lower and varied from 17% (CL) to 25% (FL). While the process of light absorption directly causes the decrease of *R_rs_* by reducing the upwelling light flux, the process of light scattering is more complex [74]. It requires the knowledge of its angular characteristics in the form of the volume scattering function. Phase functions of the volume scattering function of dispersed oils are presented in Figure 6. We also considered their spectral dependence, which is rarely practiced, although very significant (especially in optically complex waters). Scattering phase functions describe the probability of scattering an incident photon in a certain direction (scattering angle). In comparison to phase functions of natural ocean waters, phase functions obtained for dispersed oils (see Figure 1c and Figure 6) have a characteristic peak around 90–100°. Moreover, their values increase for large scattering angles near 180°. Phase functions for dispersed CL and FL have a similar shape for large angles, while phase functions for dispersed BD have lower values in the same angular range. This is the result of different size structure and lower refractive index of BD. 

### 3.2. Remote Sensing Reflectance of Oceanic Water Polluted by Dispersed Oils

**Figure 7:**
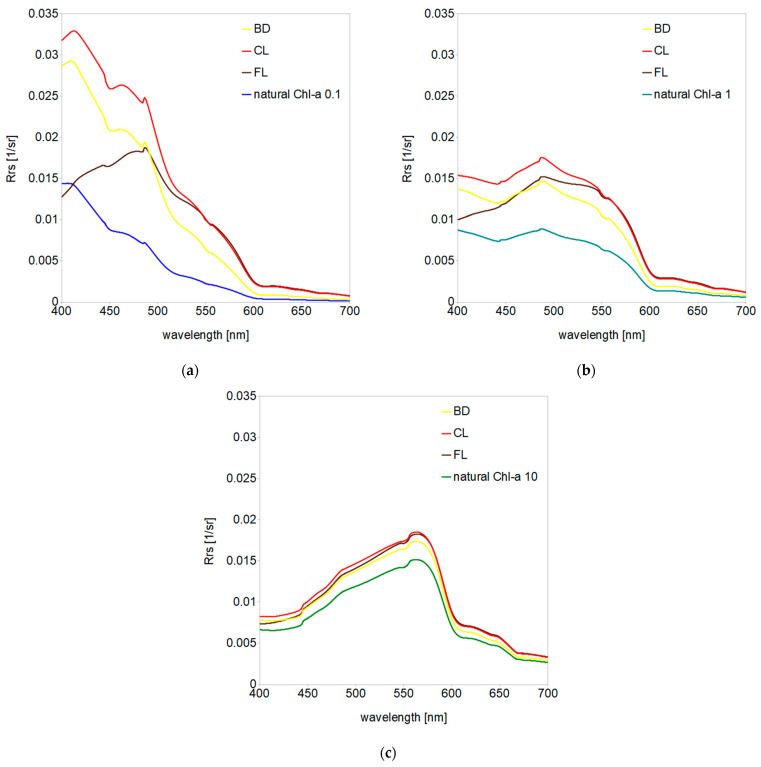
Remote sensing reflectance spectra obtained for three types of unpolluted natural ocean water models (marked by blue and green lines) and polluted by three kinds of dispersed oil: (**a**) oligotrophic ocean water characterized by *Chl-a* = 0.1 mg/m^3^, (**b**) mesotrophic ocean water characterized by *Chl-a* = 1 mg/m^3^ and (**c**) eutrophic ocean characterized by *Chl-a* = 10 mg/m^3^.

As a result of radiative transfer simulations conducted for 27 wavelengths in the visible light range (400–700 nm) for three ocean water types unpolluted and polluted by three kinds of dispersed oil, we received a set of multispectral remote sensing reflectance *R_rs_* data. In Figure 7, one can see all the obtained *R_rs_* spectra grouped by the type of ocean water and drawn with a smoothed line. 

As expected based on previous studies, the highest impact on *R_rs_* was observed for the oligotrophic ocean waters, reaching over a 2-fold increase in the blue bands of visible light spectrum, a 3–4-fold increase in the green bands and over a 5-fold increase in the red bands for CL. Interestingly, the maximal increase of *R_rs_* shifted from the blue region (~410 nm) for the oligotrophic ocean through the green region (~490 nm) for the mesotrophic ocean to the yellow region (~560 nm) for the eutrophic ocean. Because of the high shortwave absorption, dispersed crude oil FL caused significant *R_rs_* increase in the central and long wave bands, reaching 1.3-fold for eutrophic waters and 5.8-fold for oligotrophic waters. On the other hand, the presence of dispersed BD increased the *R_rs_* only 10–18% for eutrophic ocean waters, 33–66% for mesotrophic waters and up to 2.9-fold for oligotrophic waters. Table 1 illustrates a color scale of increased *R_rs_* values caused by all kinds of considered dispersed oils in all oceanic water types. While CL and FL more significantly affected the green and red bands, the maximum BD impact was placed in the green and blue bands. 

## 4. Discussion

In order to address the challenge of remote sensing of oil products dispersed in oceanic waters, we calculated some indicators routinely applied to ocean color. First, we discuss below how dispersed oil can affect remotely sensed ocean parameters in each type of oceanic water. Then, we undertake an attempt to point to the best expressions possible to differentiate oil-polluted ocean water from the unpolluted water.

### 4.1. Influence of Water Type on the Visibility of Dispersed Oil—Analysis of Ocean Color R_rs_ Band Ratios and Band Differences

The most widely used empirical algorithms for the retrieval of chlorophyll concentration in the open ocean are based on blue-to-green *R_rs_* band ratios [75,76]. They contain the ratio of the greatest *R_rs_*(*λ_blue_*) value chosen between 443 and 520 nm to *R_rs_*(*λ_green_*) closest to 555 nm. In an area contaminated with oil droplets such ratios can increase or decrease in comparison to surrounding natural seawater. Some other operational algorithms based on *R_rs_* band differences [77] can also be affected by the occurrence of dispersed oil. NASA’s standard [Chl] product merges two approaches based on the *R_rs_* band ratio and band difference [77,78].

**Figure 8:**
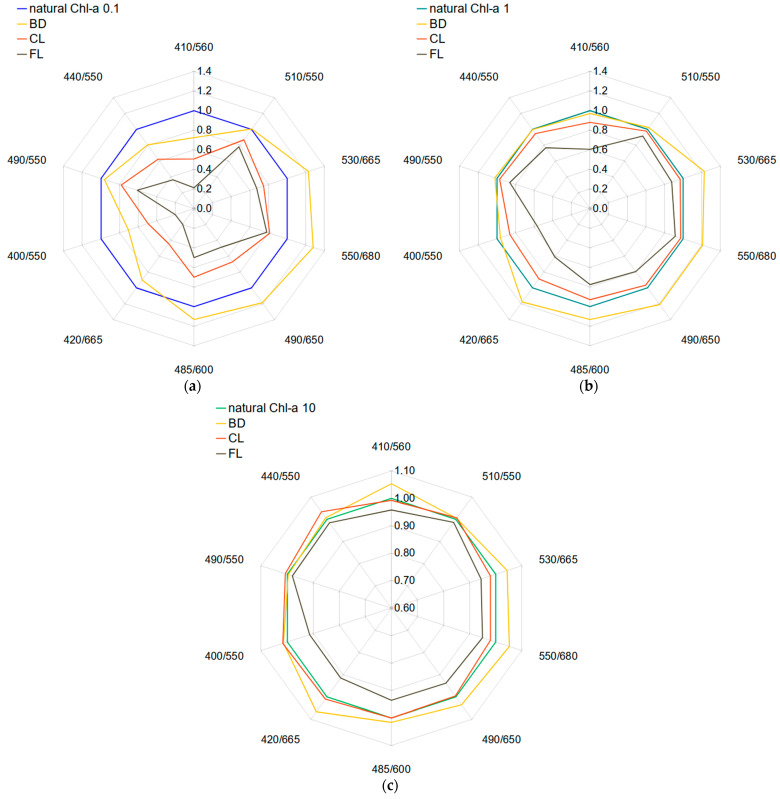
*R_rs_* band ratios for polluted water; fold change in relation to unpolluted natural water for (**a**) oligotrophic ocean water characterized by *Chl-a* = 0.1 mg/m^3^, (**b**) mesotrophic ocean water characterized by *Chl-a* = 1 mg/m^3^ and (**c**) eutrophic ocean water characterized by *Chl-a* = 10 mg/m^3^.

The impact of dispersed oils on selected *R_rs_* band ratios and band differences is shown in Table 2, where the values for oil-polluted areas are expressed as percentage difference relative to the unpolluted ones. The greatest impact of dispersed oils on these ratios was noticed on the background of oligotrophic ocean water, which is the vast majority (78%) of the global ocean, that in our paper is represented by a *Chl-a* concentration of 0,1 mg/m^3^. Ocean color *R_rs_* band ratios decreased up to 27% for dispersed BD, up to 49% for dispersed CL and up to 79% for dispersed FL. Such a decrease was a result of the addition of only 1 ppm of oil droplets present in the surface mixed layer of 30 m. Mesotrophic ocean waters, represented in our study by *Chl-a* = 1 mg/m^3^, cover areas in between continents and near-shore open ocean waters. In this type of water, only crude oil FL decreased blue-to-green ratios in a significant degree (up to 39%). Moreover, the majority of ocean color-based algorithms (e.g., NASA’s OCx) include decimal logarithms of *R_rs_* band ratios and their powers, which differ even more between natural seawater and water polluted by dispersed oil. In eutrophic ocean waters with a *Chl-a* = 10 mg/m^3^, we did not notice any substantial changes of blue-to-green *R_rs_* band ratios caused by dispersed oils; the highest was a 6% decrease noticed for FL. Polar plots of the most highly affected *R_rs_* band ratios in Figure 8 show the magnitude of their decrease or increase related to the natural ocean water.

Earlier in [48], we evaluated the possible influence of dispersed *Petrobaltic* light crude oil on some *R_rs_* band ratios in the coastal waters of the Baltic Sea. The effect on the ratio of *R_rs_(443)/R_rs_(555)* depended on the droplet size distribution and reached 18% decrease for micrometer-sized droplets characterized by the peak diameter of the log-normal function of 5 µm. Here, in the open ocean waters, biodiesel BD made a similar impact on the ratio of *R_rs_(440)/R_rs_(550)* in oligotrophic waters, and crude oil FL in mesotropic waters. Droplet size distributions measured for this study had their main maxima at 5 µm for FL and BD, and 7 µm for CL, although their shape was rather a 2- or 3-mode log-normal function than a single-mode function. In the recent study [70] conducted for dispersed light crude *Petrobaltic* and heavy crude *Romashkino* oils, Baszanowska proposed two *R_rs_* band ratios: 555/412 and 650/412 (respectively) as the best for future algorithms for dispersed oil detection. Although their conclusions were made on a different background coastal water type in the Gulf of Gdansk, and thus, they considered a much higher oil droplet concentration of 10 ppm, this is consistent with the findings of our study. Some kinds of oils tend to affect *R_rs_* blue-to-green ratios, and some others give a higher change to the red-to-blue ratios; however, these effects also depend on water type and oil droplet size distribution.

As the blue-to-green *R_rs_* ratios are dedicated to deriving bio-optical parameters of seawater, we tried to find other “unoccupied” wave bands which could be good indicators for the presence of dispersed oil. In the previous study [48], we noticed that blue-to-red *R_rs_* ratios decreased more significantly than blue-to-green ratios for micrometer-sized *Petrobaltic* oil dispersions. In this study, blue-to-red ratios (e.g., 420/665) also decreased significantly for CL and FL in oligotrophic and mesotrophic ocean waters. On the other hand, green-to-red ratios (e.g., 550/680) demonstrated their potential usefulness for dispersed BD detection indicating a 28% increase for oligotrophic waters and a 21% increase for mesotrophic waters. Similarly, the foregoing study revealed that such ratios are the best distinguishing factors for oil dispersions with a dominance of submicron oil droplets. In eutrophic waters, we did not find any *R_rs_* band ratio sensitive to dispersed oil in the concentration of 1 ppm; however, we suppose that the study performed for higher oil concentrations would reveal wavebands that are the most accurate for oil detection in chlorophyll-rich waters.

It was much easier to find *R_rs_* band differences significantly affected by dispersed oil droplets. Some red-blue differences were more than doubled in oligotrophic waters, almost doubled in mesotrophic waters and noticeably increased in eutrophic waters (see the last line in Table 2). The green-blue *R_rs_* difference increased significantly for dispersed BD in all types of ocean water and the increase predictably dropped with the growing *Chl-a* concentration. This study confirms our conclusions from previous modeling studies that *R_rs_* band differences (and their combinations) will be good candidate expressions for algorithms in the outlook of dispersed oil detection.

### 4.2. Color Index in Oligotrophic Ocean Waters

The current implementation for the NASA’s default chlorophyll algorithm employs the standard OCx band ratio algorithm merged with the color index (CI) of [77]. As described in that paper, the application of CI is restricted to relatively clear water for chlorophyll concentrations below 0.25 mg/m^3^, and is negative by definition for most clear waters. We calculated the CI for the oligotrophic ocean water model, both natural and polluted by three kinds of dispersed oils, according to the following general equation (equation 2.8 in [78]):

(1) $$\mathrm{CI}=R_{rs}\left(\lambda_{green}\right)-\left[R_{rs}\left(\lambda_{blue}\right)+\frac{\lambda_{green}-\lambda_{blue}}{\lambda_{red}-\lambda_{blue}}\cdot \left(R_{rs}\left(\lambda_{red}\right)-R_{rs}\left(\lambda_{blue}\right)\right)\right],$$

where *λ_blue_*, *λ_green_* and *λ_red_* are the instrument-specific wavelengths closest to 443, 555 and 670 nm, respectively. Obtained values of CI for different combinations of *λ_blue_*, *λ_green_* and *λ_red_* are listed in Table 3. The most affected combinations are written in bold. The presence of dispersed oil droplets significantly affected CI values. Dispersed BD nearly double reduced CI in comparison to unpolluted oligotrophic water, which means it increased the distance from *R_rs_(555)* to the linear baseline between *R_rs_(443)* and *R_rs_(670)*. As high negative CI values indicate low *Chl-a* concentrations, such a result could be misinterpreted as a much lower *Chl-a* area. Similarly, dispersed CL decreased CI for ~80%, which can lead to *Chl-a* underestimation. On the other hand, dispersed crude oil FL more than doubled CI values, which can be interpreted as a much higher *Chl-a* concentration. Hu et al. [77] noticed that *R_rs_(443)* decreases with increasing *Chl-a* values, while *R_rs_(555)* and *R_rs_(670)* remain relatively stable for oligotrophic ocean waters. Our study shows that dispersed oil pollution disrupts that dependence.

We noticed that addition of dispersed oils caused a general *R_rs_* increase in the entire visible spectral range (with some possible exceptions in the shortwave part, e.g., observed for FL). There is therefore no simple way to distinguish the presence of dispersed oil from the measured *R_rs_* spectra only. However, we strongly believe that a CI-like combination of *R_rs_* band differences and/or band ratios can lead us toward the remote detection of dispersed oil, starting from the oligotrophic water type. We assume that there is a need to create specific algorithms or weighting factors for different types of oils grouped on the basis of their IOPs and size structure. This is why the database of dispersed oil optical properties shall keep expanding.

## 5. Conclusions

In ocean waters, chlorophyll-a concentration is the primary factor derived routinely from satellite remote sensing. *Chl-a* is an indicator of phytoplankton abundance and biomass, an indicator of maximum photosynthetic rate and, thus, a measure of water quality, in terms of both natural and human-induced processes including climate change and pollution. New generations of sensors involved in such measurements become more accurate and sensitive to slight changes in the upwelling light flux and then, they are combined with corresponding methodology results in useful algorithms for the retrieval of various parameters. However, until now, there have been no methods for the remote detection of dispersed forms of oil pollution, which is very significant for the life on our planet. Visibility (and thus detectability) of oil droplets depends on the knowledge of the inherent optical properties of dispersed oil as well as the translucency of seawater (connected to the concentration of its constituents). The available database concerning optical properties of pure and dispersed oils is relatively small considering the quantity of human oil consumption nowadays and the scale of human’s ecological impact. This study demonstrated the importance of collecting complete optical data for oil dispersions and at the same time searching for the rules in the interaction between oil dispersed in seawater and light scattered in such a medium. We found out that oil dispersions dominated by micrometer-sized droplets usually increase the remote sensing reflectance in comparison to unpolluted ocean water. In the clearest studied waters characterized by *Chl-a* = 0.1 mg/m^3^, the absolute values of the *R_rs_* increased 2–6 times after simulated dispersed oil pollution. Moreover, standard *R_rs_* band ratios and band differences applied in ocean color algorithms either decreased up to 27%–80%, or more than doubled, depending on oil kind. Dispersed biodiesel BD and lubricant oil CL nearly double reduced the color index, while crude oil FL more than doubled it. This shows that oligotrophic ocean areas make the best background for studies of the influence of dispersed oil on the upwelling light flux. On the other hand, we can notice that unknown dispersed oil occurrence can lead to under- or overestimation of chlorophyll concentration in oligotrophic waters.

Furthermore, we noticed that the high variability of optical properties of oils would require us to group them in classes due to some characteristics (e.g., absorption or backscattering ratio and/or droplet size distribution) in order to find the best algorithm for remote detection of dispersed oil forms. As optical studies on dispersed oil in seawater are still rare, the access to multispectral or even hyperspectral data could take the analyses to another level. In the outlook, after gathering a larger database, combinations of several *R_rs_* band differences and/or band ratios (weighted by oil-specific or seawater-specific factors) may be investigated in order to create an algorithm needed for future testing and validation as a next step in the attempt to establish routine remote dispersed oil detection.

## Figures and Tables

**Table 1:** Color scale illustration of the increase of *R_rs_* in three typically considered spectral bands of visible light obtained through radiative transfer modeling for three types of ocean waters and three kinds of dispersed oils. Blue bands: 400–510 nm, green bands: 510–590 nm, red bands: 590–700 nm.

	BD			CL			FL		
	Blue Bands	Green Bands	Red Bands	Blue Bands	Green Bands	Red Bands	Blue Bands	Green Bands	Red Bands
oligotrophic water (*Chl-a* 0.1 mg/m^3^)	~2-fold	~3-fold	~2-fold	~3-fold	~4-fold	~5-fold	~2-fold	~4-fold	~6-fold
mesotrophic water (*Chl-a* 1 mg/m^3^)	50–80%	50–80%	30–45%	80–100%	~2-fold	~2-fold	<20%	~2-fold	~2-fold
							30–45%		
							50–80%		
eutrophic water (*Chl-a* 10 mg/m^3^)	<20%	<20%	<20%	20–30%	20–30%	20–30%	<20%	20–30%	20–30%

**Table 2:** Percentage relative differences between *R_rs_* band ratios and band differences received for unpolluted ocean waters and polluted by three kinds of dispersed oils: biodiesel BD, lubricant oil CL and crude oil FL.

	Oligotrophic			Mesotrophic			Eutrophic		
	BD	CL	FL	BD	CL	FL	BD	CL	FL
***R_rs_* band ratios,** relative difference, %									
410/560	−27	−49	−79	−3	−12	−39	3	3	−6
440/550	−19	−38	−64	0	−5	−23	1	3	−2
490/550	−4	−22	−39	2	−3	−14	0	1	−2
550/680	28	−19	−22	21	−3	−8	5	−2	−5
420/665	−10	−56	−80	18	−11	−39	7	1	−9
***R_rs_* band differences,** relative difference, %									
550–440	112	132	−18	59	24	−250	15	18	22
665–440	128	174	52	66	92	46	22	28	12
680–490	174	244	161	69	97	68	17	23	15

**Table 3:** Color index (CI) values (multiplied by 10^3^) for oligotrophic ocean waters calculated for different combinations of *λ_blue_*, *λ_green_* and *λ_red_* and compared for natural unpolluted water and polluted by three kinds of dispersed oils: biodiesel BD, lubricant oil CL and crude oil FL.

Wavelengths, nm			Color Index CI × 10^3^				Relative Difference, %		
*λ_blue_*	*λ_green_*	*λ_red_*	BD	CL	FL	Natural Unpolluted Water	BD	CL	FL
440	555	670	−5.95	−5.46	0.67	−3.11	−91	−76	121
440	555	675	−6.18	−5.74	0.51	−3.21	−92	−79	116
**440**	**550**	**670**	−6.03	−5.36	0.81	−3.19	−89	−68	**125**
440	550	675	−6.25	−5.63	0.66	−3.29	−90	−71	120
445	555	670	−5.59	−5.13	0.45	−2.87	−95	−79	116
**445**	**555**	**675**	−5.82	−5.39	0.30	−2.97	**−96**	**−82**	110
445	550	670	−5.66	−5.02	0.59	−2.94	−92	−70	120
445	550	675	−5.87	−5.27	0.44	−3.04	−93	−74	114
